# Hedgehog Cholesterolysis: Specialized Gatekeeper to Oncogenic Signaling

**DOI:** 10.3390/cancers7040875

**Published:** 2015-10-14

**Authors:** Brian P. Callahan, Chunyu Wang

**Affiliations:** 1 Chemistry Department, Binghamton University 4400 Vestal Parkway East, Binghamton, NY 13902, USA; 2 Biology Department, Rensselaer Polytechnic Institute, 110 8th Street, Troy, NY 12180, USA; wangc5@rpi.edu

**Keywords:** hedgehog, autoprocessing, cholesterol, cancer, cholesterolysis, cholesteroylation, signaling

## Abstract

Discussions of therapeutic suppression of hedgehog (Hh) signaling almost exclusively focus on receptor antagonism; however, hedgehog’s biosynthesis represents a unique and potentially targetable aspect of this oncogenic signaling pathway. Here, we review a key biosynthetic step called cholesterolysis from the perspectives of structure/function and small molecule inhibition. Cholesterolysis, also called cholesteroylation, generates cholesterol-modified Hh ligand via autoprocessing of a hedgehog precursor protein. Post-translational modification by cholesterol appears to be restricted to proteins in the hedgehog family. The transformation is essential for Hh biological activity and upstream of signaling events. Despite its decisive role in generating ligand, cholesterolysis remains conspicuously unexplored as a therapeutic target.

## 1. Introduction

In general, cell/cell signaling by hedgehog (Hh) proteins is beneficial, particularly during early development [1,2,3,4,5,6,7,8]. Severe congenital malformations are linked to mutations in the three human Hh genes—Sonic, Indian, and Desert [9,10,11]. However, in the adult persistent Hh signaling has pathogenic effects in multiple cancers. Recent studies connect Hh overexpression, predominantly sonic Hh, to sporadic tumorigenesis and metastasis in prostate, breast, and lung tissue, among others [12,13,14,15,16,17,18,19]. Validation of Hh signaling as a target for anti-cancer therapy reached a milestone in 2012 with the approval of Erivedge (Vismodegib) for the treatment of advanced basal cell carcinoma [20,21,22,23]. The path of Hh research from developmental biology to approved cancer drug is a case study in basic scientific inquiry with translational purpose [24,25,26,27,28].

**Figure 1:**
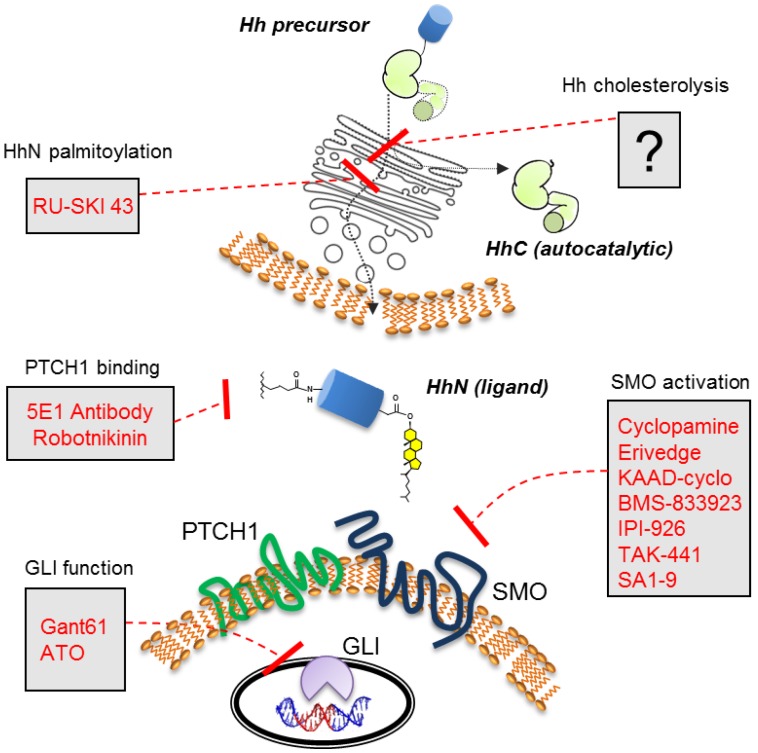
Components and selected antagonists of the Hh cell-cell signaling pathway. Hh precursor cholesterolysis occurs in the endoplasmic reticulum of the Hh expressing cell (top).

Erivedge is representative of a general class of Hh therapeutics, where the mechanism of action involves inhibiting Hh signal transduction (Figure 1). The drug interferes with a seven-pass cell surface receptor called Smoothened (SMO), the quintessential target for Hh pathway manipulation [25]. Although Hhs are not bound by SMO, SMO is required for transducing the Hh signal. The intermediary Patched (PTCH1), a 12-pass receptor, antagonizes SMO action in the absence of Hh [29,30,31,32]. When PTCH1 is bound by Hh ligand, the antagonistic interactions cease. Signal transduction events downstream of SMO1 have also ignited drug discovery efforts. The Gli1 and Gli2 transcription factors, which relay Hh signaling at the level of gene expression [33,34], have been blocked by small molecules, GANT58 and GANT61 [35]. The anticancer agent, arsenic trioxide, also seems to compromise GLI action [36], although effects vary with cell type [37]. The accompanying reviews in this issue provide timely updates in this area.

Here the spotlight shines on a less explored opportunity for pathway manipulation, Hh cholesterolysis. This unusual biosynthetic event, involving a precursor form of Hh [38], occurs upstream of Hh ligand secretion, and hence upstream of signal transduction [39,40,41,42]. Cholesterolysis represents one of two lipidation events that the Hh ligand undergoes. The other is N-linked palmitoylation [43,44,45,46]. Unlike the fatty acid modification, the catalyst for cholesterol modification is the Hh protein itself—no cofactors or accessory proteins are required. Self-catalyzed reactions like this one are classified as autoprocessing [47,48]. Mutagenesis studies indicate that diminished cholesterolysis prevents Hh ligand release, leads to premature Hh ligand degradation, and thereby stifles downstream signaling [39,41,42,49].

Small molecules that inhibit the Hh pathway in a similar manner could prove useful therapeutically, particularly in cancers driven by Hh ligand overproduction. Despite the promise, compounds that selectively inhibit cholesterolysis have not been reported. In this work, we review mechanistic details, structure/function data, along with preliminary efforts to discover cholesterolysis inhibitors. Many details are murky, and autoprocessing in general is unexplored as a therapeutic target. As a result, basic and applied research in this area remain at a formative stage since cholesterolysis was described 20 years ago.

## 2. Cholesterolysis—Liberating the Ligand

**Figure 2:**
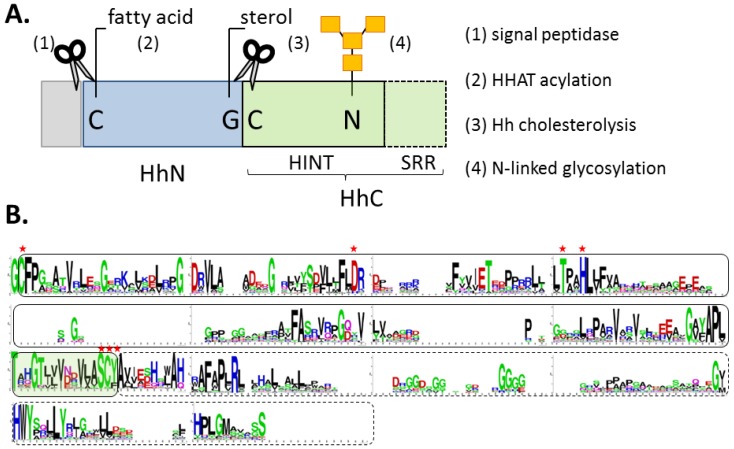
(**A**) Block diagram of Hh precursor protein, with signal peptide (grey), signaling ligand (blue), and autocatalytic segment (green). (**B**) Conserved residues in the autocatalytic segment displayed by Logoplot (http://weblogo.berkeley.edu/logo.cgi). Solid line delineates the HINT domain; hashed line, the SRR region. Residues marked with red asterisks are required for cholesterolysis.

Hh proteins are expressed as bifunctional precursors, consisting of the signaling ligand (HhN, 20 kDa) fused to a C-terminal autocatalytic element (HhC, 26 kDa) (Figure 2A). Early studies demonstrated that Hh precursor is short lived in eukaryotic cells, undergoing site-specific cleavage to separate HhN from HhC [39,40]. Beachy and his group revealed two surprising features of this reaction: first, the transformation can be reconstituted *in vitro* in the absence of accessory proteins; second, the terminal nucleophile that cleaves at the HhN/HhC junction is a sterol [38,39,50]. Cholesterol proved to be most active in the reconstitution experiments, and presumably represents the native substrate in mammals. Thus, a new post-translational modification was discovered that proceeds by peptide bond cholesterolysis [38,51]. Remarkably, two decades from those seminal publications, members of the Hh family still seem to retain “exclusive rights” to this chemistry [53].

Results of truncation experiments indicate that all catalytic activity essential for cholesterolysis resides in HhC; the N-terminal signaling domain is a bystander [38,53]. HhN can be replaced by arbitrary proteins and peptides without appreciable loss of activity [42,49,54]. HhC exhibits a bipartite organization with a hedgehog/intein domain “HINT” followed by a hydrophobic sterol recognition region, SRR (Figure 2A,B). High-resolution structural data of these segments could be invaluable; however, studies on HhC are incomplete (more below). We lack structural data for a Hh precursor, an intact HhC segment, and a HINT domain from a vertebrate. An atomic structure of the SRR region would be particularly advantageous for understanding the cholesterolysis mechanism and guiding inhibitor development.

**Figure 3:**
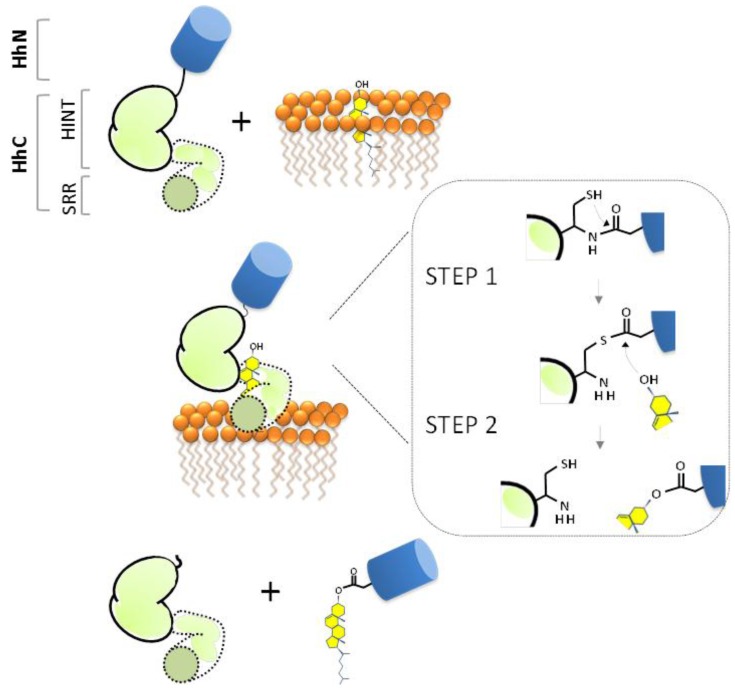
Proposed mechanism of Hh precursor cholesterolysis as a self-catalyzed event. Inset depicts the two chemical steps: an N-S acyl shift (Step 1) followed by transesterification (Step 2). Signaling ligand, HhN (blue); autocatalytic segment, HhC (green).

The standard mechanism of Hh cholesterolysis involves two steps and requires both the HINT and SRR segments [38,53,55]. As depicted in Figure 3 (Step 1), the sequence begins with the generation of an internal thioester via rearrangement of the backbone peptide bond linking HhN to HhC. The first residue of HhC, invariably cysteine, serves as the nucleophile. It seems conceivable that the scissile amide is strained to facilitate this endergonic rearrangement [56], as has been suggested in related autoprocessing reactions [57,58,59]. Mutagenesis studies have revealed several conserved residues in the HINT domain that are required for the N-S acyl shift [39,42,53]. Mechanistic roles are considered below. In Step 2 of cholesterolysis (Figure 3, Step 2), the thioester linking HhN to HhC is resolved by transesterification to cholesterol. This step liberates HhN from HhC and covalently links the newly formed C-terminus of HhN to substrate cholesterol. Deletion mapping indicate that Step 2 requires the SRR segment, comprising the last ~70 residues of HhC [53]. The source of cholesterol, its binding interactions, and the means by which its C3 hydroxyl group (pK_a_ > 15) is activated remain obscure.

### 2.1. The HINT Domain from Drosophila Melanogaster Hh Protein

**Figure 4:**
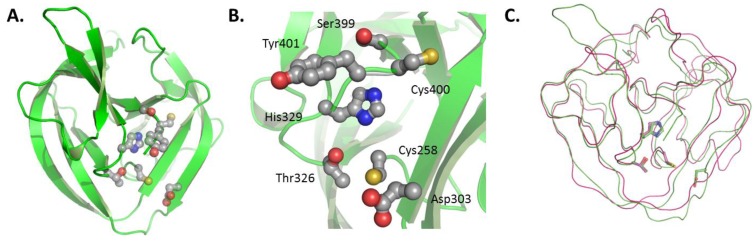
(**A**) HINT domain of Drosophila melanogaster Hh precursor (PDB#, 1AT0); (**B**) conserved catalytic residues of the HINT domain; an (**C**) alignment of Hh HINT domain with self-splicing intein (PDB#, 2IN0). Figures rendered using PyMol (DeLano Scientific LLC, Palo Alto, CA, USA).

The first, and so far only, structure relevant to HhC is that of a HINT domain reported by Hall *et al.* in 1997 [53]. The domain belongs to the *Drosophila melanogaster* (Dme) Hh precursor. It is competent to self-catalyze the first step of cholesterolysis, N-S acyl shift, but not the second. Thus, the domain can generate an internal thioester, as apparent from cleavage at the N-terminal HINT junction by water (hydrolysis) and added hydroxylamine (hydroxyaminolysis); however, activity with cholesterol is absent. The HINT domain is predominately β-strand, folded into two symmetrical lobes resembling a baseball catcher’s glove (Figure 4A). Active site residues are arranged in the glove’s pocket (Figure 4B). Striking homology exists between the HINT structure and the self-splicing domain of inteins, pointing to a common ancestor (Figure 4C) [47,53,60]. Catalytic residues in common between Hh HINT and inteins comprise the N-terminal cysteine, a conserved TXXH motif, and second cysteine at the C-terminal end of the domain. Evolutionary divergence is apparent in, for example, an active-site aspartate (Asp303 in full length *Dme* Hh numbering).

Residual activity of point mutants has helped define mechanistic roles for conserved HINT residues. Mutation of the threonine or histidine residues in the TXXH motif of the HINT compromises N-S acyl shift activity. The threonine is arranged similarly to the corresponding residue in inteins, hydrogen bonding to the nucleophilic cysteine residue (Cys258 in Dme Hh) [61,62]. This Thr residue may have a role in positioning Cys258 for attack and the leaving group, glycine, for departure. The histidine (His329) of the conserved TXXH motif may act as a general base to activate the cysteine for N-S acyl shift [55,63], or promote N-S acyl shift through ground-state destabilization [58,64]. In contrast, mutating the Hh-specific aspartate (Asp303) to alanine inhibits transesterification (Step 2) but not N-S acyl shift, suggesting a role for this residue in coupling the two steps in Hh autoprocessing [53]. Interestingly, an aspartate residue of inteins plays a similar coordinating role for self-splicing [63,65]. The downstream cysteine of the HINT (Cys400) is also intriguing, as it seems to form an intramolecular disulfide bond with Cys258 [42]; recalling the redox regulation of some inteins [59,66,67]. Mutations of Cys400 of HINT domain inhibit Step 1 and Step 2 of cholesterolysis [42].

### 2.2. Biological Role of Hh Ligand Cholesteroylation

**Figure 5:**
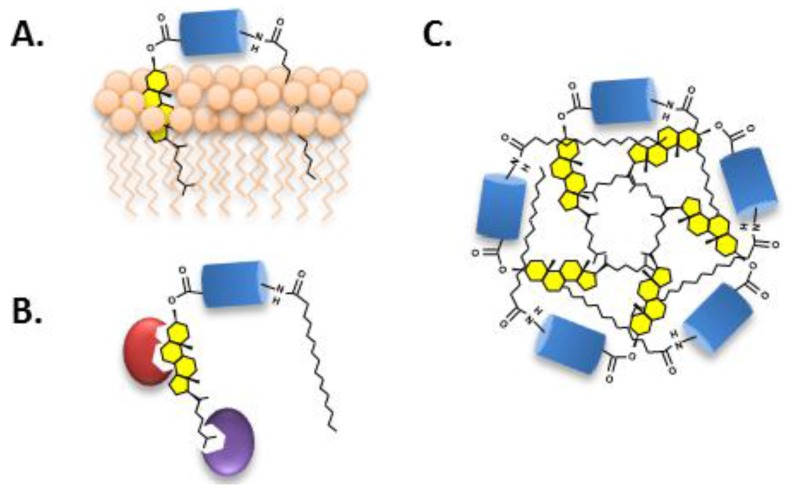
(**A**) Cholesterol targets soluble Hh ligand to cell membranes; (**B**) Proteins associate with Hh ligand via cholesterol; and (**C**) Micellization of the Hh ligand stabilized by hydrophobic interactions of the attached lipids.

Cholesterol serves as more than just a means for freeing up Hh ligand (HhN) [68,69,70]. Intuitively, covalent modification of soluble protein with cholesterol (LogP ~7) is expected to confer affinity for cellular membranes (Figure 5A). This expectation is borne out in multiple experiments, not only with Hh [71,72,73,74] but also with engineered, cholesterol-modified proteins and peptides [75,76,77,78,79]. Biophysical experiments suggest that membrane partitioning of cholesterol-modified peptides is quasi-irreversible, with half-times for dissociation approaching 3 h [75]. Avid membrane binding may explain the >10 fold greater potency in cell signaling assays of cholesterol-modified HhN compared with unmodified HhN [80,81]. It also helps rationalize why a specialized secretion mechanism is needed to release Hh ligand from the producing cell. Two proteins, Scube and Dispatched, collaborate in this role [49,82]. Cholesterol provides the handle for interactions, which effectively solubilize the Hh ligand [49] (Figure 5B). Other studies suggest that cholesterol leads to association of HhN with caveolin [83] and with lipoproteins [84], influencing intracellular trafficking and extracellular dispersal, respectively.

When the hydrophobic anchor of HhN is not bound by membrane or by protein, it may bind itself to form micelle-like structures. The proposed structure of these soluble aggregates places HhN as the “head group” with attached lipids forming a hydrophobic core (Figure 5C) [85,86]. Supramolecular structures were suggested by the anomalously large apparent molecular weight of Hh ligand interpolated by size exclusion chromatography (exp, 20 KDa; obs, >200 kDa) [52,71,87,88]. An alternative interpretation of these high molecular forms of Hh ligand has also been proposed [89].

In summary, cholesterol furnishes a membrane anchor, a point of interaction for noncovalent protein association, in addition to promoting ligand self-assembly through a hydrophobic effect. These varied effects of cholesterol on Hh ligand function likely underpin acute developmental anomalies exhibited by humans expressing cholesterolysis-defective Hh proteins [9,90], and transgenic animals expressing engineered, cholesterol-free versions of the ligand [72,91].

## 3. Targeting Cholesterolysis—A Ligand Deprivation Approach for Hh Ligand Driven Cancers

Cholesterolysis affords an attractive target in the pathway to mitigate oncogenic ligand-driven Hh signaling. Aberrant pathway activity can involve paracrine communication from tumor-to-tumor or from tumor-to-stroma; as well as tumor self-signaling through an autocrine pathway [19,92]. In these scenarios, depleting Hh ligand is expected to restrain and in some cases reverse tumor growth. Prostate cancer provides an instructive example (for reviews, see [12,93,94]). Here, two-way communication develops between transformed epithelial cells, serving as the Hh ligand producers, and benign stromal cells, the Hh responders [95]. Paracrine signaling may explain why xenografts of prostate tumors grow at accelerated rates when overexpressing Sonic Hh [96]. In addition, antibodies against Hh ligand suppress growth of primary prostate tumors and prostate cell lines [97]. The oncogenic signaling can also be diminished to some extent with SMO antagonists in animals bearing prostate tumor xenografts [98,99]. 

Recent evidence suggests that stromal cells secrete growth factors in response to Hh stimulation [100,101]; a paracrine effect replicated in controlled co-culture experiments [102]. While the reciprocal signal(s) sent from the stroma remains to be identified, several candidates exist [103]. In addition, Hh ligand released from transformed prostate epithelial cells can activate steroidogenesis in neighboring stromal cells, creating a microenvironment hospitable for tumor growth [104,105]. Crosstalk between Hh signaling and steroidogenesis is a running theme [106], as pituitary cancer and ovarian cancer exhibit similar behavior [107,108,109]. In prostate cancer, given the oncogenic activity of androgens [110], linking Hh signaling to steroidogenesis seems especially pertinent to understanding disease progression.

### 3.1. Target Attributes that Mitigate Risk

There are several attributes of cholesterolysis that garner attention for therapeutic targeting: (1) its decisive role in generating Hh ligand; (2) its position in the pathway upstream of signaling; and (3) the apparent specificity of cholesterolysis to the Hh family. These three aspects of the reaction are considered below. 

#### 3.1.1. Decisive Role Played by Cholesterolysis in Hh Signaling

The importance of cholesterolysis for Hh function emerged from genotypic studies that linked a severe congenital malformation of the brain to mutations in human *sonic hedgehog* exon 3, encoding HhC [9]. Analogous brain malformations had also been observed in patients carrying mutations in exon 1 and 2, encoding HhN [10]. That correspondence indicated that mutations in HhN can be phenocopied by mutations in HhC. Mechanistic data to connect genotype with phenotype followed from cellular studies of cholesterolysis-defective Hh precursor proteins. Guy examined the fate of Hh precursor protein that failed to react with cholesterol due to site-directed mutation or acute sterol depletion [41]. In both instances, Hh precursor remained associated with producing cells, with consequent loss of paracrine signaling [41].

More recent work by Salic and his coworkers underscored and expanded on the imperative of cholesterolysis for Hh function [42]. Among their results, the cellular site of cholesterolysis was localized to the endoplasmic reticulum (ER). Further, a retention mechanism was proposed that prevents HhC *as well as cholesterolysis-defective Hh precursor* from advancing from the ER to the Golgi. Sequestered in the ER, Hh is poly-ubiqutinated by ERAD, the organelle’s protein surveillance system [111]. HhC and cholesterolysis-defective precursor are shuttled by ERAD to the cytosol for proteasomal degradation. This transformation removes signaling ligand irreversibly. Surveillance by ERAD is sufficiently robust, the authors speculated that HhC “never leaves the ER and is not secreted.” Some evidence does exist to support limited cell-contact mediated signaling by cholesterolysis-defective precursor [112,113]. Nonetheless, the studies outlined above, and others, support the notion that blocking cholesterolysis stops *paracrine* Hh cell signaling, the pathway most closely linked to cancer.

#### 3.1.2. Upstream Target in the Pathway

Canonical signal transduction begins when secreted Hh ligand is bound by its cell surface receptor, PTCH1. With few exceptions, antagonists target events downstream of this event. In the absence of activating mutations in SMO or GLI, ligand depletion should stifle pathway activity before signaling starts and then amplifies. The monoclonal antibody 5E1, which complexes with Hh ligand in a manner that prevents interaction with PTCH1 [114], provides proof of concept [97,115]. A more upstream target, the Hh palmitoyl transferase HHAT [46], has also been targeted with small molecules by Resh and her colleagues [116]. RU-SKI-43 inhibits autocrine and paracrine signaling, and has shown exciting activity against cancers of the breast and pancreas [117,118].

Cholesterolysis appears to be the most upstream transformation targeted to date. The reaction occurs very early in the secretory pathway [41,42]. Cellular studies with brefeldin A, a fungal compound that inhibits ER to Golgi protein transport, support this notion [42]. At concentrations of brefeldin A sufficient to cause disappearance of the Golgi, Hh cholesterolysis proceeds unabated. Additional evidence for ER localization includes the involvement of ERAD in Hh degradation and association of Hh with PDI, an oxidoreductase residing in the ER [42,119,120]. Thus, cholesterolysis occurs early in the secretory pathway, soon after Hh precursor translation, and well before the first signaling event.

#### 3.1.3. A Transformation Unique to Hedgehog

Since the original reporting of Hh modification by cholesterol, attempts have been made to uncover examples of this lipidation outside the Hh family; none have been identified [121]. Proteomic screens have found numerous proteins that interact with sterols [122,123]; however, all seem to interact with this abundant lipid noncovalently. Sequence searches using human Sonic HhC turn up few hits outside the paralogues, Indian Hh and Desert Hh. Cholesterol binding proteins (enzymes, transporters, toxins, sensors) are absent from the results of our database searches, suggesting that HhC harbors a unique scaffold for sterol interaction. This aspect bodes well for the development of selective Hh inhibitors. Finally, we note that the homology of the three human HhC segments raises the possibility of designing a single cholesterolysis inhibitor active against all three human Hh precursors. A broad-spectrum antagonist could prove crucial to combat functional compensation, which has been seen in transgenic knockout mice [124].

### 3.2. Assays for Cholesterolysis Amenable to High Throughput Screening

To facilitate screening of chemical libraries for potential Hh cholesterolysis inhibitors, we as well as others have introduced optical activity assays suitable for microplate readers. These assays replace the conventional means of assaying Hh cholesterolysis by sodium dodecyl sulfate-polyacrylamide gel electrophoresis (SDS-PAGE). Because throughput is low and kinetic analyses are discontinuous, SDS-PAGE is not considered a viable platform for chemical screening campaigns. The newer assay systems employ fluorescent labels to monitor cholesterolysis continuously in multi-well plates, improving accuracy and throughput.

**Figure 6:**
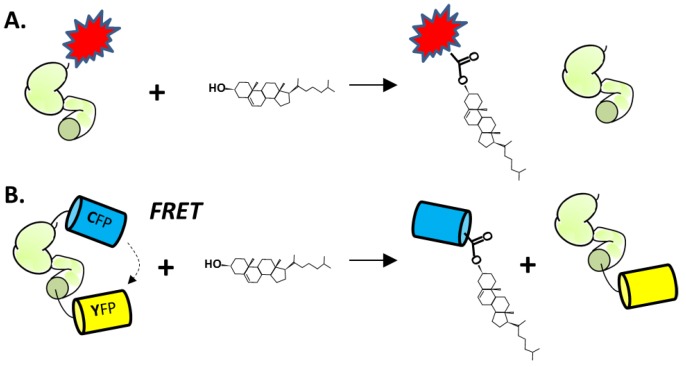
(**A**) Cholesterolysis assay using HhC (green) fused to the FLaSH, peptide-dye complex (red). (**B**) Cholesterolysis assay using HhC fused to cyan and yellow fluorescent proteins.

The first microplate assay makes use of changes in fluorescent polarization (FP) that accompany cholesterolysis of an engineered Hh precursor protein [125]. The construct consists of HhC from Drosophila melanogaster that is equipped with an N-terminal fluorescent FlAsh-tag (Figure 6A). The FlAsh-tag replaces the Hh ligand sequence (HhN). As mentioned above, HhN is not essential for HhC activity. Cholesterolysis of the engineered precursor liberates the FLAsh tag peptide, resulting in a decrease in fluorescence anisotropy that can be measured by FP. This assay was used to screen 446 compounds in the NIH Clinical Collection, resulting in the identification of two potential inhibitors, Zafirlukast (PubChem CID, 5717) and Honokiol (PubChem CID, 72303). Larger screening efforts have not been reported. Although attractive in many respects, the FP assay does require refolding of the precursor construct, labeling with arsenic-based reagents, and suffers from a small dynamic range.

More recently, Owen *et al.* described an optical cholesterolysis assay that employs Förster resonance energy transfer (FRET) [126]. An engineered fluorescent Hh precursor is again utilized, except that the labels are soluble recombinant proteins. The key construct, termed C-H-Y, is composed of cyan (C) and yellow (Y) fluorescent proteins (CFP and YFP, respectively) joined to the amino and carboxyl termini of HhC (Figure 6B). Adding cholesterol to buffered solutions of purified C-H-Y, leads to a fourfold attenuation of the FRET ratio, consistent with liberation of CFP from the precursor by substrate cholesterol. Kinetics of C-H-Y cholesterolysis at pH 7.1, the prevailing pH of the endoplasmic reticulum (ER) [127], are remarkably similar to processing measured by pulse-chase experiments in cultured mammalian cells (0.0006 s^−1^) [41]. The observed rate is also within range of autocatalytic “protein splicing” reactions brought about by inteins [128,129,130,131], as discussed in Section 2.1.

The FRET precursor C-H-Y serves double duty, providing both HTS-compatible activity assay, while also reporting on small-molecule binding to HhC. The latter feature was uncovered in studies with the Hh cholesterolysis inhibitor, phenyl arsine oxide (PhAsIII) (PubChem CID, 4778) [132]. When added to solutions containing C-H-Y, PhAsIII induced quenching of the construct’s FRET, suggesting a change in protein conformation. NMR analysis identified multiple conserved residues at the active site of HhC that are bound by PhAsIII, including two cysteine residues (Cys303 and Cys400). Accordingly, no FRET quenching was observed in control experiments with a C303A C-H-Y mutant, where the nucleophilic cysteine of HhC is replaced by alanine. In a pilot small molecule screen using C-H-Y, we observed similar quenching by a second compound, butyl 3,5-dinitro-4-(1-phenyltetrazol-5-yl)sulfanylbenzoate (PubChem CID 4358899), that inhibits cholesterolysis irreversibly. The binding site of this latter compound was mapped to Cys400 in HhC, a conserved cysteine residue whose mechanistic role in cholesterolysis remains enigmatic. The dual function of the FRET system as activity assay and binding reporter make it an attractive platform for high throughput screening.

### 3.3. Endogenous Regulators of Hh Cholesterolysis

Along with searches for inhibitors in libraries of synthetic compounds, it seems worthwhile to consider the possibility of endogenous regulators of Hh cholesterolysis. Recently, Xie *et al.* demonstrated that zinc, the ubiquitous divalent cation, could inhibit Hh autoprocessing *in vitro* and in cells [133]. Zinc is known to inhibit protein splicing mediated by inteins [134,135,136]. Because inteins and Hh HINT domains are homologous, it seemed plausible that zinc could also inhibit Hh autoprocessing. Indeed, Hh autoprocessing assays indicate inhibition by zinc with a K_i_ of 2 μM. Zinc also inhibits Hh autoprocessing in a cellular environment with primary rat astrocyte culture. Solution NMR revealed that zinc inhibits autoprocessing by binding to active site residues of the Hh HINT domain.

**Figure 7:**
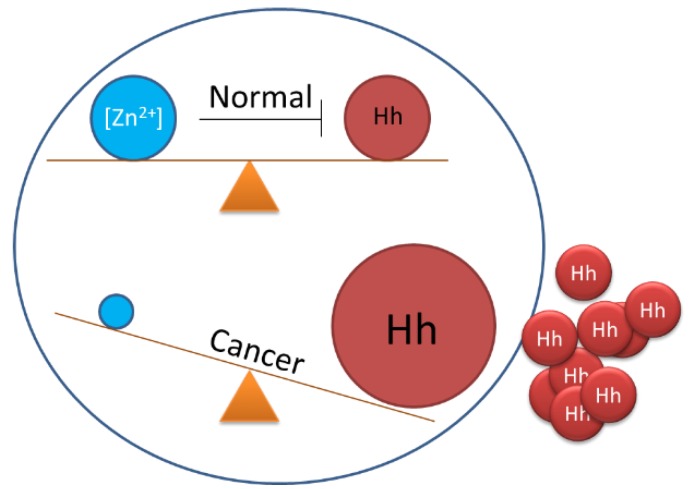
Proposed balancing of Hedgehog biosynthesis by cellular zinc concentrations and disruption in cancer.

Zinc deficiency is found in many types of cancer, including prostate, lung and ovarian cancer [137,138]. In these three cancers, Hh ligand is overproduced resulting in abnormal activation of Hh signaling pathway [97,139,140,141,142,143,144]. The data by Xie *et al*. suggests that there is a mechanistic link between zinc deficiency and Hh-ligand dependent activation of the Hh signaling pathway [133]. Thus, low zinc concentration in tissues can deregulate Hh autoprocessing, boosting Hh ligand production. The zinc/Hh axis (Figure 7) is now being investigated in these cancers. 

## 4. Conclusions and Outlook

It seems certain that no single target will suffice for blocking every type of Hh-driven malignancy. Antagonists of the receptor Smoothened (SMO), for example, can generate dramatic remissions, yet efficacy can also be compromised by SMO mutation [145], and against tumors driven by the Hh ligand, responses have been disappointing [146]. Cholesterolysis offers an alternative target for intervention. It supplies a transformation that is early in the pathway, essential, and uniquely hedgehog. Devising selective cholesterolysis inhibitors represents one of several outstanding challenges for this ligand deprivation approach (Table 1). Here again though, cholesterolysis inhibitors are expected to show therapeutic limits, particularly for ligand-independent Hh cancers. The successes and the shortcomings of Hh inhibitors, as a whole, should serve to highlight cancer’s heterogeneity and encourage bi- and tri-target treatment strategies.

**Table 1:** Areas for future work.

Quantify the tipping point, where physiological Hh signaling becomes oncogenic Identify small molecules that selectively inhibit Hh cholesterolysis *in vivo* Develop optical Hh cholesterolysis assays for cell studies and deep tissue imaging Determine Hh precursor protein structure at atomic resolution Understand the mechanism of deregulated Hh biosynthesis in transformed cells
